# Characterisation of Colorectal Cancer Cell Lines through Proteomic Profiling of Their Extracellular Vesicles

**DOI:** 10.3390/proteomes11010003

**Published:** 2023-01-11

**Authors:** Kathleen A. Heck, Håvard T. Lindholm, Barbara Niederdorfer, Eirini Tsirvouli, Martin Kuiper, Åsmund Flobak, Astrid Lægreid, Liv Thommesen

**Affiliations:** 1 Department of Clinical and Molecular Medicine, Norwegian University of Science and Technology, 7491 Trondheim, Norway; 2 Department of Biology, Norwegian University of Science and Technology, 7491 Trondheim, Norway; 3 The Cancer Clinic, St. Olav’s University Hospital, 7030 Trondheim, Norway; 4 Department of Biomedical Laboratory Science, Norwegian University of Science and Technology, 7491 Trondheim, Norway

**Keywords:** extracellular vesicles, sEVs, intracellular signalling, proteomics, phosphoproteomics, colorectal cancer

## Abstract

Colorectal cancer (CRC) is one of the most prevalent cancers, driven by several factors including deregulations in intracellular signalling pathways. Small extracellular vesicles (sEVs) are nanosized protein-packaged particles released from cells, which are present in liquid biopsies. Here, we characterised the proteome landscape of sEVs and their cells of origin in three CRC cell lines HCT116, HT29 and SW620 to explore molecular traits that could be exploited as cancer biomarker candidates and how intracellular signalling can be assessed by sEV analysis instead of directly obtaining the cell of origin itself. Our findings revealed that sEV cargo clearly reflects its cell of origin with proteins of the PI3K-AKT pathway highly represented in sEVs. Proteins known to be involved in CRC were detected in both cells and sEVs including KRAS, ARAF, mTOR, PDPK1 and MAPK1, while TGFB1 and TGFBR2, known to be key players in epithelial cancer carcinogenesis, were found to be enriched in sEVs. Furthermore, the phosphopeptide-enriched profiling of cell lysates demonstrated a distinct pattern between cell lines and highlighted potential phosphoproteomic targets to be investigated in sEVs. The total proteomic and phosphoproteomics profiles described in the current work can serve as a source to identify candidates for cancer biomarkers that can potentially be assessed from liquid biopsies.

## 1. Introduction 

Colorectal cancer (CRC) is one of the world’s most prevalent cancers with an estimated 1.4 million new cases diagnosed annually. CRC is described as a “silent” disease, and few patients with CRC exhibit clinical symptoms until late stages of the disease when the cancer has metastasised to other organs in the body [1]. CRC develops in a multistep process involving the disruption of biological events driving cellular proliferation, differentiation and apoptosis. Molecular mechanisms underlying these processes, which include the mitogen-activated protein kinase (MAPK), the phosphatidylinositol 3-kinase (PI3K)/protein kinase B (AKT), the transforming growth factor beta (TGF-β), p53, mTOR and the Wnt signalling pathways, are often disrupted due to mutations or the altered activity of proteins [2].

Small extracellular vesicles (sEVs), ranging from 50–200 nm in size, are secreted from cells in both physiological and pathological conditions [3]. They can be isolated from various biofluids including blood, urine and saliva, which makes them attractive compared to current invasive diagnostic procedures. These vesicles have been found to carry nucleic acids, lipids and proteins such as receptors, enzymes and transcription factors representative of their cell of origin. sEVs were reported to play a key role in cellular communication through the transfer of biological signals between cells over long distances throughout the body, and have been found to contribute to numerous physiological and pathological processes including cancer development and metastasis as well as drug resistance [4,5] through influencing intracellular signalling cascades in recipient cells [6,7,8]. Several studies have shown that in hypoxic conditions occurring in the tumour microenvironment higher levels of sEVs were excreted from the tumour. These in turn have been demonstrated to promote a proliferative effect through the activation of the Wnt signalling pathway, contributing to increased angiogenesis within the tumour microenvironment [9,10,11,12]. Luchetti et al. found that the release of sEVs from a CRC cell line could promote cancer cell migration through the enhanced phosphorylation of signalling molecules, such as SRC and ERK, with a subsequent activation of the MAPK signalling cascade [13]. Peinado et al. demonstrated how MET oncoprotein enriched in sEVs from melanoma cells altered the tumour microenvironment by promoting a metastatic niche through the activation of downstream mediators of MET signalling through the phosphorylation of S6-kinase and MAPK1 [14]. These defining characteristics of sEVs, together with their high biological stability, contribute to their emergence as a source of biomarkers in cancer.

Post-translational modifications (PTMs), such as phosphorylation, are well-described events governing intracellular signalling, which are known to be central in cancer biology. The phosphorylation status of proteins is frequently altered due to protein mutations [15,16,17]. Gaining insight into the phosphoproteomic pattern of diseases such as CRC might therefore lead to the development of improved diagnostics [18]. Cancer-cell-derived sEV cargo has been found to comprise, e.g., phosphorylated receptor tyrosine kinases including EGFR, HER2 and C-MET, crucial in driving intracellular signalling resulting in cellular proliferation and expansion as well as metastasis and angiogenesis [14,19]. 

The aim of this study was to examine to what extent the proteomic content of sEVs reflect their paternal cell and whether the proteome of sEVs would uncover features of cancer cells that could allow for improved insight into cellular processes such as disease-associated signalling aberrations. We additionally wanted to uncover activated signalling pathways or molecular signatures through global phosphoproteomics analysis of whole-cell lysates. 

To investigate these questions, we selected the commonly used colorectal cancer cell lines HT29, HCT116 and SW620. SW620 is isolated from a metastasised colon cancer while HT29 and HCT116 are isolated from the colon. Some of the well-described pathogenic mutations found in these cell lines are as follows: SW620 contains KRAS, APC and TP53; HCT116 contains BRCA2, CTNNB1, KRAS, PIK3CA and TGFBR2; and HT29 contains APC, BRAF, SMAD4 and TP53 [20].

Our investigation detected a broad overlap of proteins packaged in sEVs compared to their cell of origin. We identified characteristic protein expression patterns for the cell lines known to be key players in cellular signalling cascades including MAPK, Wnt, TGF-β, p53 and mTOR signalling and further investigated these through phosphoproteomic analysis of CRC cells. A subset of proteins involved in the PI3K-AKT signalling pathway was enriched in sEVs. Further investigation into CRC-biology-associated proteins reveals TGF-β signalling proteins, TGFB1 and TGF-β receptor 2 (TGFBR2), to be enriched in sEVs only. sEV proteomic cargo was also found to contain several specific proteins playing key roles in intracellular signalling. These results may allow for a better understanding of sEVs in CRC cell biology and serve as a source to investigate candidates for cancer biomarkers.

## 2. Materials and Methods

### 2.1. Cell Culture

Human colorectal carcinoma cells HCT116, HT29 and SW620 were directly acquired from the National Cancer Institute (NCI) via the NCI-DCTD repository in Frederick, MD (MTA #1-5578-17). No cell line identity verification was performed in house, but the NCI characterises the cell lines using ~120,000,000 SNP arrays and oligonucleotide-based HLA typing as well as other approaches [21]. The cells were cultured with a complete Gibco™ RPMI 1640 medium supplemented with 10% EV-depleted foetal bovine serum (FBS; Gibco, New York, NY, USA) and 1% penicillin/streptomycin (Gibco, New York, NY, USA). The cells were incubated at 37 °C in a 5% CO_2_ air atmosphere.

### 2.2. sEV Lysate Isolation and Preparation

HCT116 (5 × 10^6^), HT29 (8 × 10^6^) and SW620 (5 × 10^6^) cells were seeded and cultured in a 23 mL medium (175 cm^2^ flask) for 72 hrs to allow sEV secretion. The average number of cells from a 1xT175 flask was 25 × 10^6^ (HCT116), 28.5 × 10^6^ (HT29) and 42 × 10^6^ (SW620), respectively. The media were collected (eight flasks per cell line) and centrifuged at 500× *g* for 5 min to remove cellular debris. The sEVs were isolated from the eight flasks using a standardised ultracentrifugation protocol as described by Peinado et al. [14] A Beckman Coulter Optima™ XE-90 ultracentrifuge (Brea, CA, USA) was employed. Larger EVs were removed following an initial 20 min 12,000× *g* ultracentrifugation. The sEV fraction collected was further isolated by ultracentrifuging at 100,000× *g* for 70 min. One isolation resulted in an sEV fraction, which was then washed in either (i) 21 mL PBS for nanoparticle tracking analysis (NTA) or (ii) an added 200 µL of 2% paraformaldehyde for transmission electron microscopy (TEM) analysis before (iii) suspension in a 20 μL lysis buffer (Thermo Scientific Halt™ Protease & Phosphatase Single Use Inhibitor Cocktail (100×) in Thermo Scientific Pierce™ RIPA Buffer). Next, the sEVs were gently rocked at 4 °C for 3 h before measuring the protein concentration using a Bio-Rad assay for proteomic analysis. All of the above work was carried out at 4 °C cooled settings. 

### 2.3. Cell Lysate Preparation and Protein Measurement

Cell lysates for all three cell lines were collected simultaneously as the media were removed for sEV isolation. For cell lysis, media were removed and the cells were washed with cold PBS, trypsinised and then centrifuged at 500× *g* for 5 min. The supernatant was removed and the cells were resuspended in cold PBS before centrifuging once again at 800 RPM for 8 min prior to resuspension in a 300 uL lysis buffer (Thermo Scientific Halt™ Protease & Phosphatase Single Use Inhibitor Cocktail (100×) in Thermo Scientific Pierce™ RIPA Buffer). Cell lysates were continuously swayed at 4 °C for a period of 3 h before measuring protein concentration using Bio-Rad assay (Bio-Rad Laboratories, Hercules, CA, USA).

### 2.4. Particle Size Determination

The sEV pellets were suspended in PBS 1:500 and examined using the NanoSight NS300 analyser allowing the particles to fall in the instrumental linear range of detection, which is approximately 1 × 10^8^ to 1 × 10^9^ particles/mL. Triplicate video recordings (60 s) were obtained and analysed by the NTA software (KX-NS300 SX/BX/ML/XE Version 7.0 e, Malvern Panalytical, Malvern, United Kingdom). Mean particle numbers/mL of 2.56 × 10^8^ ± 4.92 × 10^7^ particles/mL (HCT116), 158 nm 8.06 × 10^8^ ± 3.34 × 10^7^ particles/mL (HT29) and 2.12 × 10^9^ ± 1.67 × 10^8^ particles/mL (SW620) were acquired. The parameters for detection included a detection threshold equal to 5 and camera level equal to 10 as per the NanoSight NS300 for all measurements. For TEM examination, the sEVs were resuspended and fixed in cold 2% paraformaldehyde in PBS. Droplets of sEVs were put on Formvar-carbon coated copper grids (200 mesh) for 5 min, fixed in 2.5% Glutaraldehyde (GA) in 0.1 M Sorensen’s phosphate buffer for 10 min, washed in H_2_O reverse osmosis water, stained and embedded in 2% uranyl acetate (UA) for 10 min. The excess liquid on the grids was removed using filter paper and then air-dried. The grids were examined using a transmission electron microscope (JSM-1011 TEM, JEOL), at 100,000× magnification and a voltage of 80 kV. Images were captured with a Morada digital camera with iTEM software—BoRAS.

### 2.5. Immunoblotting

**Figure 1:**
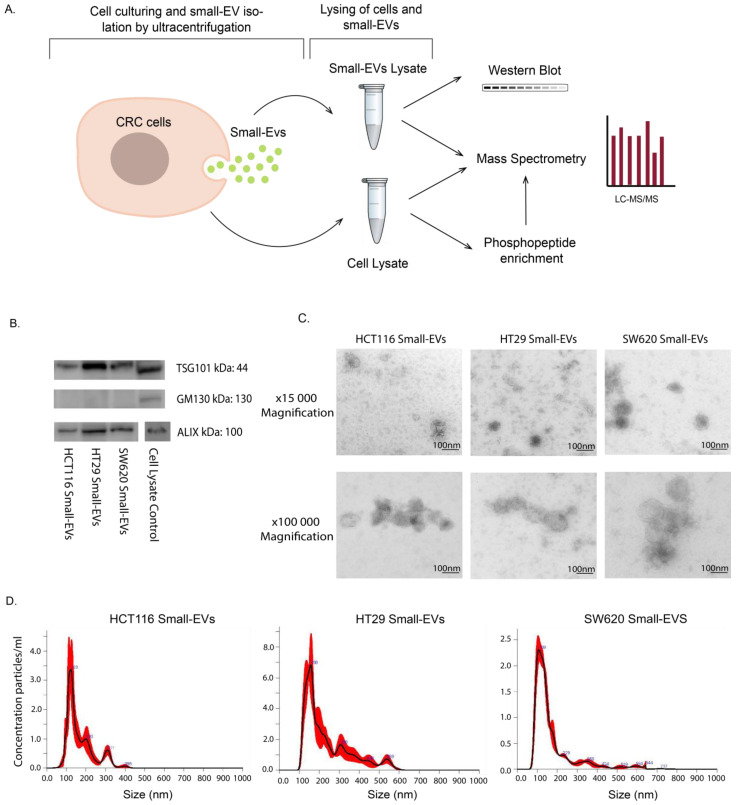
Experimental setup and small-EV characterisation for HCT116, HT29 and SW620 CRC cell lines. (**A**) Lysates of whole cells and small EVs isolated by ultracentrifugation were subjected to analysis by Western blot, direct mass spectrometry (MS) proteomic analysis and phosphopeptide enrichment followed by MS phosphoproteomic analysis. All experimental and analytical work was conducted with three biological replicates. (**B**) Western blot of small-EV positive biomarkers (ALIX, TSG101) and negative biomarker GM130. Loading quantities and original blots are specified in Supplementary Figure S1. (**C**) Transmission electron microscopy (TEM) of small EVs at 15,000 and 100,000 magnifications with scale bar of 100 nm. (**D**) Nanosight (NTA) analysis showing particle size distribution of small EVs in all cell lines.

Equal amounts of protein from both the cellular and sEV lysates were loaded into 4–12% Bis-Tris gels (the protein concentrations are specified in Figure 1 and in supplementary data in Supplementary Figure S1). The proteins were next transferred onto polyvinylidene fluoride (PVDF) membranes for 50 min at 200 V at 4 °C. The membranes were blocked in 5% bovine serum albumin (BSA) diluted in tris-buffer saline with 0.5% Tween 20 for 1 h at room temperature with constant rocking. The membranes were incubated with primary antibodies: ALIX (1:1000 CST, Cat: 2171, BioNordika, Oslo, Norway), TSG101 (1:1000 Abcam, Cat: ab30871, Cambridge, UK) and GM130 (1:500 BD Biosciences, Cat: 610823, San Jose, CA, USA). The antibody binding was detected using HRP-linked secondary antibodies according to the manufacturer’s instructions (Dako). All the above antibodies were diluted in 5% BSA solution except ALIX, which was diluted in 5% non-fat milk.

### 2.6. Mass Spectrometry Experimental Setup

All the experimental conditions for mass spectrometry were run with three biological replicates and one technical replicate. For shotgun analysis, 50 µg of protein extract was reduced, alkylated and digested on magnetic HILIC beads as described by the producer (Resyn Biosciences, Pretoria, South Africa). For phosphoproteomic analysis, 500 µg of protein extract was reduced, alkylated and digested prior to phosphopeptide enrichment using TiIMAC magnetic beads according to the protocol (Resyn Biosciences, South Africa). Following desalting using C18 StageTips [22], liquids were removed from the peptides using a SpeedVac centrifuge and 50 µL of 0.1% formic acid was used for resuspension. The LC-MS/MS platform used consisted of a QExactive HF orbitrap mass spectrometer (Thermo Fisher Scientific) interfaced with an Easy-nLC 1000 UHPLC system (Thermo Fisher Scientific) via a nanospray ESI ion source (Proxeon, Odense). A C-18 trap column (Acclaim PepMap100, 75 μm i. d. × 2 cm, C18, 3 μm, 100 Å, Thermo Fisher Scientific) and a C-18 analytical column (Acclaim PepMap100, 75 μm i. d. × 50 cm, C18, 2 μm, 100 Å, Thermo Fisher Scientific) were used for peptide separation. Buffer A (0.1% formic acid) and buffer B (CH3CN, 0.1% formic acid) with a gradient were used in the column separation. A flow rate of 250 nL/min was used with the following settings: from 5% to 40% B in 165 min, 40–80% B in 10 min and 80% B in 5 min. A QExactive HF mass spectrometer was used to analyse eluted peptides using the following parameters: 1E5 for MS/MS scans, automatic gain control target value of 3E6 for Orbitrap MS and HCD fragmentation with normalised collision energy 29 and Electrospray voltage 1.9 kV. Each MS scan (*m*/*z* 400–1800) was acquired at a resolution of 12,000 FWHM, followed by 15 MS/MS scans triggered for AGC targets above 2E3, at a maximum ion injection time of 50 ms for MS and 100 ms for MS/MS scans.

### 2.7. Mass Spectrometry Analysis

The software Thermo Scientific™ Proteome Discoverer™ (http://www.thermoscientific.com/content/tfs/en/product/proteome-discoverer-software.html, accessed on 1 September 2022) version 2.3 (PD) was used to quantify protein levels from the raw MS data. The raw files were inspected to determine optimal search criteria using Preview version 2.3.5 from Protein Metrics Incorporate (https://pubs.acs.org/doi/abs/10.1021/ac200609a, accessed on 1 September 2022).The following parameters for the search were used: precursor mass tolerance of 10 PPM and fragment mass tolerance of 0.02 Dalton; phosphorylation of Serine/Threonine/Tyrosine, acetylation of Protein N-terminal, oxidation of Methionine and deamidation of Asparagine/Glutamine as a dynamic post-translational modification and carbamidomethylation of Cysteine as static; enzyme specified as Trypsin with a maximum of two missed cleavages allowed. Only unique peptides with high confidence were used for the final protein group identification by setting downstream analysis of the peptide spectra matches (PSM) and false discovery rate (FDR) for both protein and peptide to 1%. The integration of the area under the peak curve was used to calculate abundance. Each protein group abundance was normalised by the total abundance of all identified peptides at FDR <1%. The summed up median values for all unique peptide ion abundances were mapped to respective proteins using the label-free quantification algorithm (http://www.mcponline.org/content/13/9/2513.long, accessed on 1 September 2022). The resulting values were scaled for all averages with the Precursor Ion Quantifier node (http://tools.thermofisher.com/content/sfs/posters/PN-64857-LC-MS-LFQ-Proteome-Discoverer-IMSC2016-PN64857-EN.pdf, accessed on 1 September 2022) for MQ and PD, respectively. The values for all samples were log2-transformed and subjected to principal component analysis (PCA). In order to calculate phospho-site occupancies, PTM was set to True in the MQ experiment design for all the enriched shotgun proteomics samples.

### 2.8. Differentially Expressed Protein Identification

In order to identify the differentially expressed phospho-enriched peptides among the three cell lines, a differential expression analysis (DEA) of the data was carried out using the R package DEP [23]. Prior to the DEA, all the potential contaminants identified during the MS analysis were removed. Furthermore, as some peptides were not quantified in all replicates, an additional filtering step for proteins that were identified in two out of three replicates of at least one condition was performed. For the identification of the differentially expressed peptides, linear models combined with empirical Bayes statistics were employed and all possible comparisons between cell lines were generated. The differentially expressed peptides were assessed with a false discovery rate (FDR) < 0.01. The most significantly differentially expressed phospho-enriched peptides in the triplicate whole-cell samples in all cell lines were visualised as a heat map. 

### 2.9. Data Visualisation

HUGO Gene Nomenclature Committee (HGNC) gene symbols were associated with the accession numbers of proteins from the shotgun proteomics analysis. Proteins without the HGNC gene symbol were filtered out as these accession numbers are outdated or belong to other species. Out of 7306 unique accession numbers, 95 *Bos taurus* proteins, 42 *Homo sapiens* proteins and 9 *Mus musculus* proteins were filtered out. Venn diagrams were created with the R-package eulerr [24] and heat maps were created with the R-package pheatmap [25].

## 3. Results

### 3.1. Proteomic Analysis of CRC Cells and sEVs 

This study aimed to characterise and compare the proteomic signatures and signalling configurations in sEV cargo and cells of origin for three CRC cell lines: HCT116, HT29 and SW620 (Figure 1A). The EV samples, isolated from culture media by ultracentrifugation, were characterised by Western blot analysis, transmission electron microscopy (TEM) and nanosight tracking analysis (NTA). We showed that EVs from all three cell lines contained the EV-associated markers ALIX (PDCD6IP) and TSG101, while they were negative for the cis-Golgi membrane protein GM130 (Golgin subfamily A member 2), confirming that our EV preparations were not contaminated with cellular components (Figure 1B and Figure S1). Furthermore, we also confirmed that all common EV marker proteins as defined by Hoshino et al. are present in our mass spectrometry data presented below (Figure S1C) [26]. The EV particles were found to be of typical sEV size and morphology (Figure 1C) with mean size distributions of 123 nm (HCT116), 158 nm (HT29) and 109 nm (SW620) (Figure 1D), corresponding to well-known sEVs characteristics [27]. Together these results confirm the successful isolation of sEVs from our cell line culture media.

**Figure 2:**
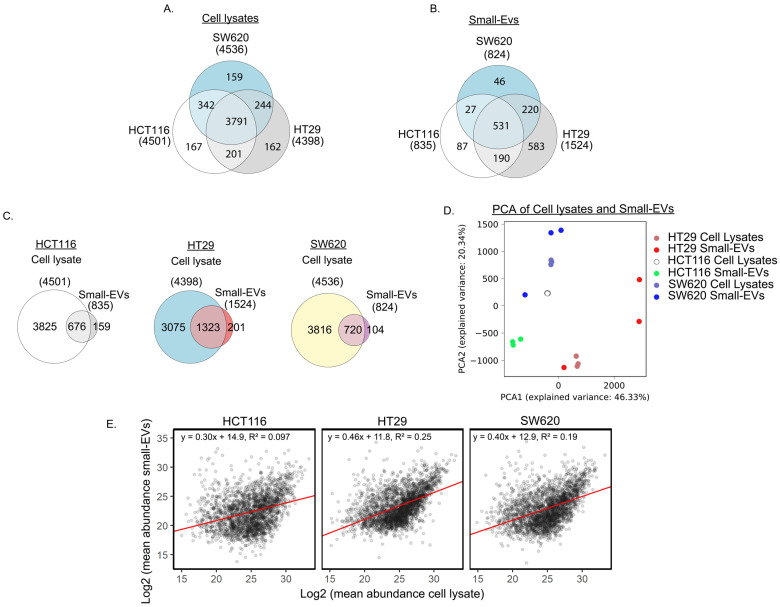
Proteomic analysis of cell lysates and small EVs from three CRC cell lines. (**A**) Venn diagrams comparing cell lysate proteins detected in at least two biological replicates per cell line. (**B**) Comparison of proteins detected in at least two biological small EV replicates per cell line. (**C**) Venn diagrams of individual CRC cell line proteins detected in at least two replicates of cell lysates and small EVs. (**D**) Principal component analysis (PCA) based on proteins detected in both cells and small EVs for the three biological replicates in all three cell lines. (**E**) Comparison of mean abundance of proteins in cell lysate and in small EVs.

To characterise their proteomic content, lysates from whole cells and sEVs from the three CRC cell lines HCT116, HT29 and SW620 in three biological replicates were subjected to LC-MS/MS analysis. We noted that similar numbers of proteins, approximately 4500, were detected in each of the CRC whole-cell preparations (Figure 2A). Abundances can be found in Table S1. As expected, most of the proteins were observed in all three whole-cell lysates, while, for each cell line, approximately 160 proteins were detected only in this individual cell line. Principal component analysis (PCA) and clustering of Pearson correlation between samples revealed that each of the cell lines displays a distinct proteomic profile, with good agreement between the three replicas (Figure S2A,B). 

When comparing the proteome of the sEVs from the three cell lines (Figure 2B), we observed that approximately the same number of proteins was detected in HCT116 and SW620, 835 and 824, respectively, whereas nearly two-fold more proteins (1524) were detected in HT29 sEVs. Abundances can be found and summarised in Supplementary Table S2. We observed an overlap of 531 proteins in sEVs from all three cell lines, leaving a modest number of proteins that were observed only in HCT116 or SW620 sEVs while 583 proteins were observed only in HT29 small EVs. Our observations of the diverging number of proteins detected in sEVs released from the three cell lines is similar to the proteomic heterogeneity between sEVs from different cell lines reported by others [28,29]. PCA and clustering of Pearson correlation between samples of the sEV proteomes displayed a distinct profile with good agreement between the three replicas from each cell line (Figure S2A,C). Investigation of Gene Ontology (GO) annotations of the 531 proteins observed in sEVs from all three cell lines (Table S3) showed that the clusters with the highest enrichment score encompassed GO terms associated with cell adhesion, cell translation and GTP/GDP signal transduction activity as well as the Wnt, TNF-β, MAPK and NIK/NF-κB signalling pathways [30]. The enrichment in the Wnt and NIK/NF-κB signalling pathways include proteasome proteins that regulate the activity of these pathways. All 21 proteins enriched in NIK/NF-κB signalling are involved in the proteasome complex as well as a large portion of the Wnt pathway proteins. 

Next, we compared the proteomic content of sEVs to the proteins extracted from their cells of origin. Most of the sEV proteins were also detected in their whole-cell counterpart, with an overlap of 81% in HCT116 and 87% in HT29 and in SW620 (Figure 2C). Interestingly, over 100 proteins in HCT116 or SW620 sEVs and approximately 200 proteins in HT29 sEVs were not detected in their cells of origin, possibly indicating an enrichment of specific proteins in sEVs.

To further assess the similarity between the sEVs and their cell of origin, we performed PCA analysis on proteins detected both in sEVs and in cells. This analysis revealed that the sEV profiles are more similar to their cell of origin than to other sEV profiles or other cell lines (Figure 2D). The variability between sEV biological replicas is larger than the variation between whole-cell proteomic profiles. Although the replicates from both HT29 and SW620 showed one sEV sample markedly different from the other two replicates, all replicates still remain relatively similar to their cell line of origin. To assess the direct similarity between protein levels detected in sEVs and cell lysates we plotted these values against each other (Figure 2E). This plot reveals that there is some, but not a perfect, correlation between protein levels in small EVs and cell lysates. Taken together, our analysis indicates that the proteomic content of our sEV samples mirrors that of the cells they are derived from, which is promising for future diagnostic use.

**Figure 3:**
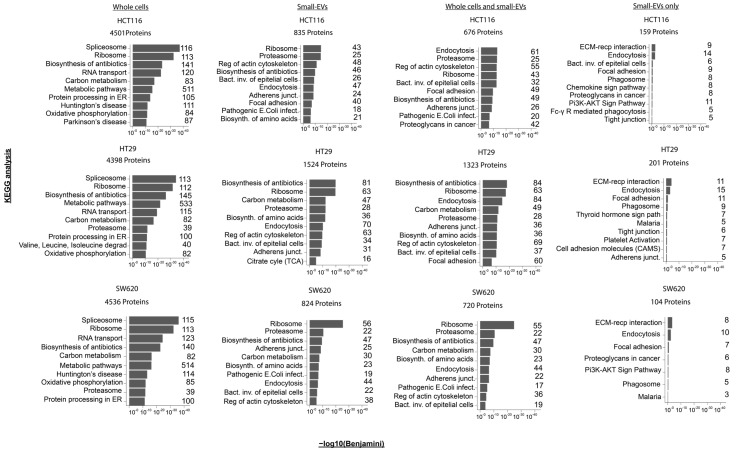
Pathway enrichments in proteomes of whole-cell and small-EV lysates. For each of the CRC cell lines, KEGG pathway enrichment analysis was performed for the mass spectrometry-derived proteome from (i) whole cells, (ii) small-EV lysates or (iii) for the proteins observed in both the whole-cell and small-EV preparations or (iv) proteins observed only in the small EVs (based on Figure 2C). The top 10 most significant KEGG terms are shown above where the *x*-axis represents the −log10 (Benjamini corrected *p*-value). The number of proteins implicated in the individual KEGG term following analysis is listed to the right-hand side of each bar chart. All analyses were performed using the David Bioinformatics Resources 6.8 tool (https://david.ncifcrf.gov/, accessed on 1 September 2022).

In order to further investigate functional aspects of the whole-cell and sEV proteomes, we performed KEGG pathway enrichment analysis using DAVID GO term analysis of the following four different sets of proteins: (i) all proteins from whole cells, (ii) all sEV proteins, (iii) proteins observed in both whole cells and sEVs and (iv) proteins observed in sEVs only (all results shown in Supplementary Table S4). We noticed that, within each of the protein sets, the enrichment patterns are very similar across the three cell lines, with similar top 10 significantly enriched KEGG pathways and similar protein numbers observed for each of the pathways (Figure 3). The whole-cell proteome showed an enrichment for proteins involved in spliceosome, ribosome and several metabolic processes as well as in protein processing within the endoplasmic reticulum (ER). The sEVs proteome is distinct from the whole-cell proteome and displays a larger functional variation between cell lines, even though ribosome-pathway-associated proteins are also prominent in two of the three cell lines (HCT116 and SW620). In the sEVs preparations, proteins associated with endocytosis and the regulation of actin cytoskeleton are among the top 10 KEGG terms ordered by significance. The set of proteins observed in both cells and specific sEVs also display an enrichment of proteins involved in endocytosis and the regulation of the actin cytoskeleton along with proteins associated with functions in adherence junctions. Proteins observed only in sEVs showed enrichment for ECM–receptor interaction and endocytosis—complying with the well-established generation of sEVs via the endosomal pathway (Figure 3 and Supplementary Table S4). Interestingly, proteins involved in PI3K-AKT signalling were among the top 10 enriched KEGG pathways proteins in sEV-specific proteins (Figure 3). A similar result was found using the STRING enrichment tool for the KEGG pathway, where the PI3K-AKT pathway was significantly enriched in sEV-specific proteins in HCT116 and SW620 with FDRs equal to 0.01 and 0.035, respectively, see Supplementary Table S5. The PI3K-AKT pathway, along with the MAPK, TGF-β and Wnt signalling pathways, is known to be deregulated in CRC [2,31]. We were therefore intrigued to take a closer look at the expression patterns of proteins in whole cells and sEVs known to participate in the PI3K-AKT signalling pathway and of general importance to colorectal cancer biology. 

**Figure 4:**
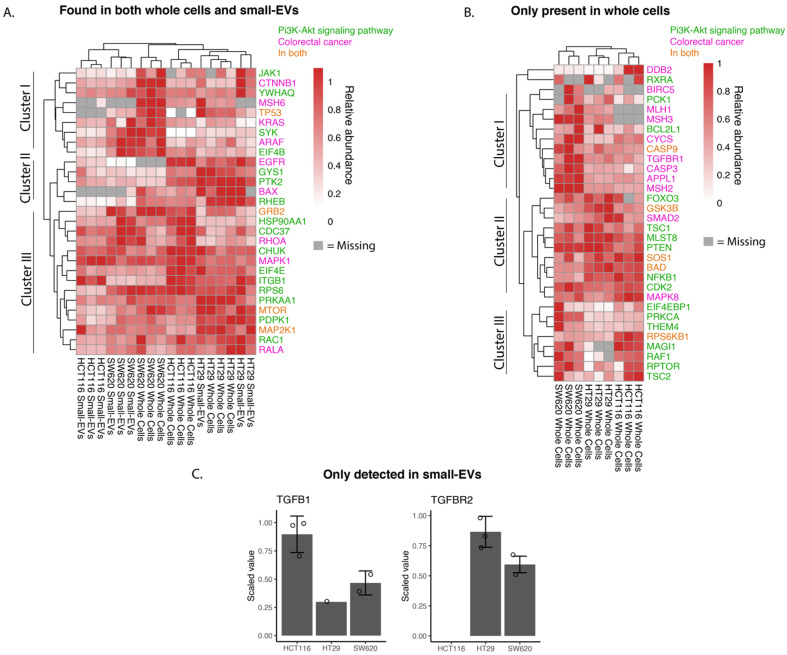
Abundance of whole-cell and small-EV proteins involved in colorectal cancer and PI3K-AKT pathways. Shotgun proteomic data for small-EV and whole-cell biological replicates highlighting proteins present in CRC, hsa05210 and proteins related to PI3K-AKT signalling, hsa04151, as per the KEGG database. (**A**) Proteins found in both whole-cell and small EV lysates. (**B**) Proteins only found in whole-cell lysates. (**C**) Proteins only found in small-EV lysates displayed in a bar chart with detected values shown with circles. Relative abundances are scaled to the sample with maximum abundance for small EVs and cells individually for each protein. Gray colour in the heat maps represents proteins not detected in the sample and only proteins with at least three detected values across all samples are shown.

We investigated the relative abundance of proteins implicated in the KEGG pathways “Colorectal Cancer” (hsa05210) or “PI3K-AKT pathway” (hsa04151) as depicted in Figure 4. For most of the whole-cell and sEV proteins, there is a good correlation of observed protein levels across the three biological replicates. We note that for SW620 and HT29 the expression patterns of the sEV proteins in the chosen pathways show strong similarities to the patterns in whole cells, while HCT116 sEV samples cluster somewhat further away from their parental whole-cell samples (Figure 4A). 

Cluster analysis of abundancies of proteins participating in the chosen pathways and observed in both whole cells and small EVs identified in three main clusters (Figure 4A). Proteins in cluster I are more highly expressed in SW620 whole cells and their sEVs compared to the other two cell lines and are implicated in Wnt signalling (e.g., β-catenin, CTNNB1), P53 signalling (TP53) and MAPK signalling (KRAS and ARAF). HCT116 whole cells and sEVs, however, showed little to no expression of TP53 and KRAS compared to the other two cell lines. The proteins of cluster II are more highly expressed in HT29 whole cells and sEVs with key proteins involved in mTOR signalling (RHEB—GTP-binding protein) and MAPK signalling activation (PTK2—Focal adhesion kinase 1). Epidermal growth factor receptor (EGFR), a receptor tyrosine kinase initiating intracellular signalling, could be detected in whole-cell lysates in HCT116 and HT29 cell lines. Cluster III captured proteins detected at relatively high abundance in all samples and encompassed proteins known to play a role in the MAPK signalling cascade, such as MAPK1 (ERK2), MAP2K1 (MEK1) and GRB2 (Growth factor receptor-bound protein 2), the mTOR signalling pathway including mTOR (Serine/threonine-protein kinase mTOR) and the NFκB signalling pathway protein (CHUK, Inhibitor of nuclear factor kappa-B kinase subunit alpha). In summary, the findings presented in the heat map of Figure 4A indicate potential differences in individual cell line tumorigenesis and the possible heterogeneity of sEV packaging between cell lines.

From the heat map analysis of “Colorectal Cancer” and “PI3K-AKT”pathways proteins detected in whole-cell lysates only (Figure 4B), we comment on the three main clusters observed. Cluster I is characterised by proteins expressed at higher levels in the metastatic SW620 cells compared to the other two cell lines and encompasses proteins involved in the DNA mismatch repair (MMR) system (MLH1, MSH3 and MSH2), TGF-β receptor 1 (TGFBR1). In clusters II and III, we find proteins driving key signalling cascades including the MAPK (Sons of Sevenless—SOS1, RAF proto-oncogene serine—RAF1), Wnt (Glycogen synthase kinase-3 beta—GSK3B), mTOR (Tuberin—TSC2, Regulatory-associated protein of mTOR—RPTOR) and NFκB signalling pathways (Nuclear factor NF-kappa-B p105 subunit—NFKB1). While the expression levels of cluster II proteins appear uniformly high across the three cell lines, the cluster III proteins display higher levels in SW620 and HCT116 cells compared to HT29 cells. We can again observe from this heat map differing proteomic signatures between the cell lines driving CRC (Figure 4B).

TGFB1 and TGFBR2 were detected only in sEV lysates and not in any of the whole-cell lysates (Figure 4C). The TGFBR1 and TGFBR2 complex initiates signalling when interacting with TGFB1. Both receptor proteins have previously been identified in sEVs (ExoCarta.org); however, they have not been identified specifically in CRC sEVs as per the database. It is of interest to note that high levels of TGFB1 and TGFBR2 are associated with the increased proliferation and inhibition of apoptosis in CRC cells [32,33]. 

Taken together, we conclude that the proteomic data displayed in Figure 4 indicate that whereas cell-line-specific proteomic traits are replicated in their respective sEVs, some proteins implicated in CRC may be exclusively detected in sEVs. 

### 3.2. The HCT116, HT29 and SW620 Phosphoproteome 

The activity of a protein is not only affected by its abundance but is also dependent on its phosphorylation state. To complement our previous proteomics data, we therefore investigated the phosphoproteomic profiles of the cell lysates from the three CRC cell lines included in this study with three biological replicates for each cell line. Together, this gives a broader view of the status of these cells. 

**Figure 5:**
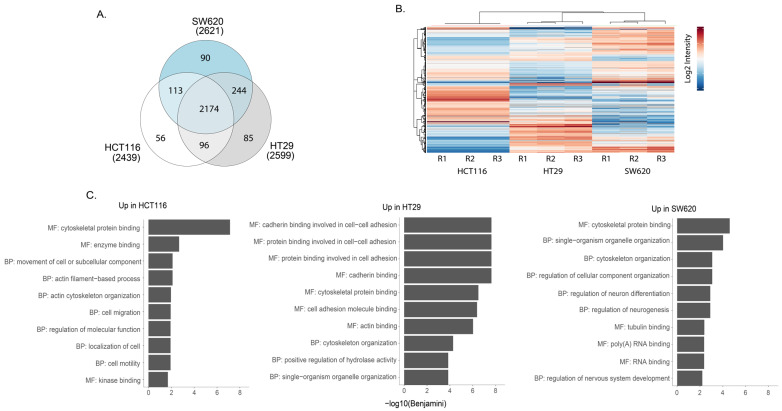
Phosphoproteomic analyses of HCT116, HT29 and SW620 cells. (**A**) Venn diagram comparing the phospho-enriched proteomic content of cell lines. (**B**) Heat map presenting the most statistically significant differentially expressed phospho-enriched proteins in whole cells. R1, R2 and R3 represent three independent biological replicates. (**C**) Top 10 GO terms associated with proteins from differentially expressed proteins upregulated in indicated cell line compared to other two cell lines (i.e., Up in HCT116 = upregulated in HCT116 vs. HT29 + upregulated in HCT116 vs. SW620) according to DAVID GO term analysis frequently asked terms (FAT). GO terms associated with BF = biological process and MF = molecular function can be seen. All BF and MF terms are found in Table S2. All analyses were performed using David Bioinformatics Resources 6.8 (https://david.ncifcrf.gov/, accessed on 1 September 2022).

Mass spectrometry analysis of phosphopeptide-enriched preparations from whole-cell lysates captured approximately 2600 phosphoproteins in each of the three cell lines. Of these, 85% were detected in all cell lines while a small subset was found in only one of the cell lines (Figure 5A). Pearson correlation show that the replicates cluster together (Figure S3). All phosphopeptide abundance data are shown in Supplementary Table S6. Heat map cluster analysis of the phosphopeptides with the highest differential expression illustrates phosphoproteomic differences between the three cell lines (Figure 5B). We undertook GO enrichment analysis of proteins represented by the phosphopeptides that seemed characteristically high in one cell line compared to the other two (all data shown in Table S7). The top 10 GO term for each comparison revealed differences between the three cell lines (Figure 5C). In the case of HCT116, a greater enrichment for GO terms proteins with tyrosine kinase activity was observed when compared against HT29 and SW620 (Table S7). Further, KEGG analysis of the differentially expressed phosphoproteins revealed the enrichment of several signalling cascades implicated in CRC (Table S8). A higher enrichment for the MAPK signalling pathway was seen in HCT116 and HT29 compared to the other cell line, while NFκB enrichment was seen in SW620 in comparison against HCT116 and HT29 cell lines accordingly (Table S8). Thus, phosphoproteomic analysis revealed specific differences between cell lines in phosphorylated proteins that reflects the activity of different signalling pathways. 

**Figure 6:**
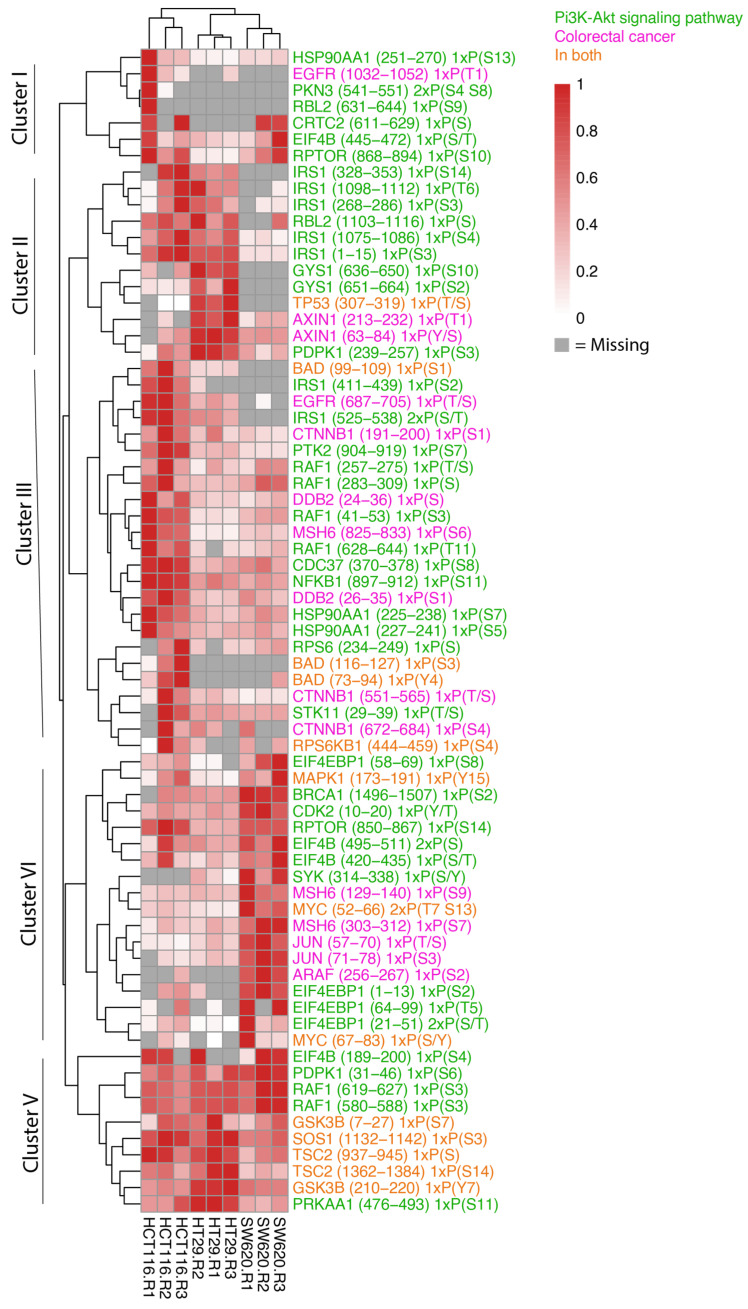
Abundance of phosphopeptides in whole-cell samples in colorectal cancer and PI3K-AKT pathways. Heat map of the phosphopeptide abundance data for whole-cell biological replicates highlighting proteins present in CRC, hsa05210 and proteins related to PI3K-AKT signalling, hsa04151, as per the KEGG database. Relative abundances are scaled to the sample with maximum abundance for each protein. Gray colour is indicative of proteins not detected in the sample and only proteins with at least three detected values are shown.

Given that we observed phosphopeptides associated with cellular processes relevant for CRC, we were next interested in taking a closer look at the phosphorylation patterns of proteins known to play a role in the proteins represented in the KEGG pathways “Colorectal Cancer” (hsa05210) or “PI3K-AKT pathway” (hsa04151) (Figure 6). The heat map of these phosphopeptides shows striking cell-line-specific protein phosphorylation patterns that can be assigned to five main clusters. 

Most phosphopeptides in cluster I display very low reproducibility between the biological replicates. However, RPTOR serine (S) (868–894), known for its role in mTOR signalling, and EIF4B S/Threonine (T) (445–472), which plays a role in protein transcription, appeared across all three cell types with highest expression noted in HCT116 and SW620 cells. Cluster II identifies phosphopeptides that display higher abundance in in both HCT116 and HT29 cells or in only HT29 cells compared to SW620. HT29 cells encompass greater levels of TP53 T/S (307–319) and PDPK1 S (239–257) phosphopeptides where a high total protein abundance is also observed in Figure 4A. In addition, the phosphopeptide and Wnt signalling protein Axin (T (213–232) and S (63–84)) is noted in both HT29 and SW620 cells although it is most highly expressed in HT29. A key component downstream of the insulin receptor is the insulin receptor substrate 1 (IRS1), which plays a key role in driving pathways including MAPK and PI3K-AKT. We observe high levels of IRS1 phosphopeptides in HCT116 cells and markedly lower levels in HT29. The IRS1 phosphosites include IRS1 S (1098–1112) encompassing phosphosite S1101, which blocks downstream activation of the AKT-PKB pathway when phosphorylated [34]. Cluster III and IV identified highly expressed phosphopeptides in HCT116 cells (cluster III) and SW620 (cluster IV) compared to the other cell lines encompassing intracellular signalling components involved in multiple signalling pathways previously detected (Figure 4A,B). These include phosphopeptides within the MAPK pathway (EGFR T/S (687–705), RAF1 T/S (257–275), PTK2 S (904–919), MAPK1 tyrosine (Y) (173–191), ARAF (S (256–267), MYC S/T (52–66) and S/Y (67–83)), Wnt signalling (CTNNB1 S (551–565)) and S (672–684)), mTOR pathway (RPTOR S (850–867)), and NFκB signalling (NFKB1 S (897–912)). Cluster V comprises phosphopeptides present at high levels in all cell lines. The cluster includes signalling phosphopeptides detected within our proteomic data such as RAF1 S (619–627), GSK3B Y (210–220), TSC2 S (1362–1384) and SOS1 S (1132–1142). HT29 and HCT116 cells displayed higher levels of GSK3B, TSC2 and SOS1 compared to SW620 while RAF1 was seen in greater abundance in SW620 cells.

Taken together, our data show that the cell lines studied here display phosphorylation statuses that are relevant in cancer and highlight the individual phosphorylation states of the CRC cell lines used in this study. 

## 4. Discussion

Colorectal cancer is one of the most prevalent cancers worldwide. Currently, the clinical management of colorectal cancer is directed by the histological and molecular analysis of biopsies of tumours. Extracting biopsies can be invasive and measurements originating from a small part of a tumour do not necessarily reflect the overall state of the disease. sEVs are a potential source of disease-associated biomarkers without any of these issues. They can be isolated from biofluids, which are easier to acquire than biopsies, and small EVs from biofluids originate from the whole tumour [35]. Extensive research has revealed that other components of body fluids such as circulating tumour DNA and circulating tumour cells can be useful (“liquid biopsies”) to understand the phenotypic and genetic heterogeneity of cancer and sEVs might add to this repertoire, capturing a separate set of information about the state of the cancer. However, if sEVs are to be used as biomarkers it must be established whether they represent their cells of origin and if the proteins that are packaged in sEVs are potentially interesting targets for biomarker analysis. 

In this study, we find that most of the proteins detected within sEVs overlap with those in their cell of origin. However, the overlap is not complete, as a subset of the proteins is only detected in sEVs and not in their cell of origin. The presence of these sEV-specific proteins indicate that proteins of certain processes are enriched in sEVs and therefore easier to detect in sEVs than in cell lysates. However, we cannot establish with certainty why these proteins were not detected in the cell lysate fraction. These proteins should be present in the cell lysate samples as the sEV proteins originate from the same cells. Studies of breast cancer cells and lung cancer cells also find that sEVs broadly reflect their cell of origin, while only a subset of the proteins is enriched in sEVs [36,37]. These results emphasise that the relationship between the abundance of a protein in a cell and in small EVs is protein- or process-dependent, and it can therefore be useful to consider datasets such as the one provided in this study when considering specific proteins from sEVs as potential biomarkers. 

The mechanisms guiding the packaging of proteins into sEVs is not completely understood, but ESCRT (endosomal sorting complexes required for transport)-dependent and independent systems, tetraspanins, and lipid-dependent mechanisms are reported to be involved [38,39]. The Characterisation of proteins found in small EVs highlights that higher-order oligomerisation and plasma membrane association predicts protein trafficking to sEVs [40]. It is therefore not surprising that proteins involved in endocytosis turned up in our GO term analysis of sEV-specific proteins. This finding is also supported by the proteomic characterisation of sEVs from tissues and cell lines, which also found the GO term endocytosis to be associated with proteins uniquely found in sEVs [26]. Our initial pathway enrichment analysis of the proteins, detected only in sEV fractions and not in whole cells, comprises many proteins involved in the PI3K-AKT signalling pathway. A possible explanation for this observation may be derived from considering known mechanisms involved in the formation and packaging of sEVs, whereby signalling proteins associated with the cell membrane, which includes PI3K-AKT proteins, would become enriched in the packaged vesicle [41]. This observation highlights that it may be useful to consider the location and function of a protein when investigating whether it could serve as a potential biomarker originating from sEVs. Furthermore, the PI3K-AKT signalling pathway plays an important role in diseases such as CRC (32), indicating that the mechanism of packing proteins into sEVs does enrich for proteins relevant to diseases. Furthermore, we detected a number of proteins that are enriched specifically in sEVs and relevant in intracellular signalling pathways associated with CRC. These include MAPK [17], Wnt [42], P53 [43], mTOR [44], NFκB [45] and TGF-β [46]. sEVs derived from cancer cells have been previously studied for their proteomic content associated with these signalling cascades [47,48,49,50]. However, it is important to mention that enrichment of a GO term describing a signalling pathway does not mean that we can conclude that a signalling pathway is changed from our data, only that we detect proteins that potentially can influence these pathways. 

From the proteins annotated to the KEGG pathways colorectal cancer and PI3K-AKT signalling, one peptide of the TGF-β signalling proteins TGFB1 and TGFBR2 was detected in sEVs but not in the whole-cell lysates. Interestingly, these proteins are not listed as associated with sEVs isolated from CRC in the Exocarta.org database or found in EVs isolated from HCT116, HT29 or SW620 according to the deposited data in another study [26], emphasising that different approaches for EV isolation influences whether these proteins are detectable. Together with TGFBR1, TGFB1 and TGFBR2 are involved in initiating and driving TGF-β signalling and play key roles in driving disease including CRC [32,33]. TGF-β signalling has a dual role in both the suppression and promotion of epithelial cancer carcinogenesis through a number of processes including the inhibition of cell proliferation, induced differentiation and promoting apoptosis in normal cells and early cancer stages while also suppressing the immune system and promoting angiogenesis [51,52]. Around 20–30% of CRCs contain mutations in TGFBR2, which is especially seen in MSI CRC [53,54,55]. Interestingly, TGFBR2 was only detected in sEVs from SW620 and HT29 and absent in HCT116. This is in line with the mutational status of HCT116, which has been reported to have a lysine replaced with a serine in position 128, causing a premature stop signal giving a dysfunctional TGFBR2 [20]. However, it must be emphasised that we can only conclude that the proteomic levels of TGFBR2 are missing from our analysis, not that they are not present in the cell. A study of HCT116 cell lines with reintroduced TGFBR2 signalling found that the MSI driver mutation of TGFBR2 can influence the protein packaging of sEVs whereby a later study by the same group identified 48 small-EV-specific proteins, such as fibronectin (FN1), which plays a pro-tumorigenic role in CRC [56], to be regulated in a TGFBR2-dependent manner [53,54]. This result highlights how the enrichment of certain proteins in small EVs can give a novel view of proteins compared to the analysis of their cell of origin. Furthermore, as these results are only observed in this single mass spectrometry experiment, we hope that future studies will follow up and confirm these observations. 

Changes in the amount of a protein may not necessarily be accompanied by parallel changes in their activity status. Although we do not always know whether phosphorylation is associated with activation or inactivation, phosphorylation levels can be a more accurate measurement of protein activity compared to protein amounts. This makes the phosphorylation data included here an important reference. The phosphorylation state of our proteins showed a large overlap between our cell lines whereby less than 10% of phosphopeptides were detected exclusively in one of the cell lines. The differential expression of the HCT116, HT29 and SW620 cell lines revealed specific differences, which were further shown through GO term and KEGG pathway enrichment analysis. The differences in phosphopeptides included changes in a multitude of proteins normally associated with important changes in colorectal cancer. We found a greater abundance of phosphorylated MAPK1/ERK2 Y (173–191) in HCT116 cell lysates compared to other cell lines. This is in agreement with the cell line’s KRAS mutation, which is linked to increased ERK activity [57] and has been found enriched in HCT116 but not in HT29 and SW620 by Western blot [58]. We also noted MYC S/T (52–66) and S/Y (67–83) phosphorylation. T58, S62, S71 and S81 phosphorylation have been previously outlined in CRC where GSK3β was found to inhibit MYC phosphorylation at T58 preventing its degradation, while phosphorylation on serine 62 by ERK was found to favour the protein’s stabilisation. These denote MYC’s targeted therapeutic potential in regulating CRC cells [59]. EGFR phosphopeptide T/S (687–705) was also detected in HCT116 and HT29 cell samples, correlating with the previously identified T693 and S695 phosphosites, which are linked to the inhibition of the downstream activation of the MAPK8/JNK pathway driving RAS-MEK-ERK MAPK signalling [60]. In summary, these results highlight the validity of our data and emphasise the potential importance of these data as a reference for further studies. 

sEVs extracted from a bodily fluid do not solely originate from cancer cells, but also from other cells of the body. In this study, we therefore chose to study sEVs extracted from cell lines as we can clearly know what cells the sEV originate from. Cancer cell lines have different characteristics from primary tumours but do have cellular and molecular traits that resemble tumours [61]. The study of their molecular make up can therefore inform us about cancer cell diversity and characteristic states and assist in finding candidates for biomarkers for prognosis and choice of therapy. However, it is important to mention that this study does not prove that changes observed in sEVs from cell lines relevant for CRC would be detectable in biofluids. For the analysis of plasmatic EVs, the establishment of methodologies for purifying different subpopulations of Evs and analysing their contents easily and precisely will be required for their clinical implementation. As the development of EV research is rapid [26], we believe that Evs will be used as valuable biomarkers in the near future.

Together, our results show specific enrichment for each of our three cell lines in terms of key signalling proteins driving CRC carcinogenesis while also highlighting the whole cell’s representation within the matching sEV. They additionally confirm the value of studying the proteome within sEVs as potential sources for further biomarker research [62,63]. It is important to note, however, that there are limitations with the proteomics methods utilised here and that the absence of detected proteins does not necessarily mean that the protein is absent from the sample. In addition, a limitation of this study is that proteoformes are not evaluated in any of the analysis, meaning that the shotgun proteomic analysis, phosphoproteomic analysis and any downstream analysis are limited to the consensus protein sequence, which can represent a multitude of isoforms with different biological functions. Furthermore, the fraction of the phosphorylation of proteins being investigated may not always be captured due to low phosphorylation levels [64] and should not be taken as proof that they are absent. These individual phosphopeptide examples highlight the heterogeneity of CRC [65,66]. Our data support and offer insight into known signalling pathways in these CRC cell lines allowing for more in-depth phosphoproteomic analysis to be investigated in future studies. 

Having a broader overview of signalling pathway proteins in cells and sEV cargo allows for opportunities to further study potential CRC biomarkers and advance novel diagnostics and prognostics as well as understand potential combinatorial treatment targets to investigate. While we argue that this study presents a greater overview of signalling pathway proteins in cells and sEVs through the investigation of their proteomic landscape, we also recognise some important challenges existing still in the field of sEVs. The isolation of sEVs gives limited material, which can hinder certain types of analysis, such as phosphoproteomic analysis, which requires large amounts of input material. Furthermore, there is inherent variability in sEV proteomic isolation and the release of sEVs between cell types can vary [67,68]. However, the literature comprises several studies that have successfully obtained phosphoprotein data from cell-line-derived sEVs [14,69] as well as from liquid biopsies, including patient plasma [70] and patient urine, in which multiple samples from the patient were pooled allowing for a larger amount of sEVs to be collected [71]. These studies emphasise the value and feasibility of further pursuing sEVs for phosphoproteomic biomarkers in patient samples.

## 5. Conclusions

sEVs represent their cell of origin, and their release into biofluids makes them an attractive diagnostic target. Here, we have used proteomics to globally compare the content of sEVs to their parental cell lysates in three CRC cell lines. We found an association between proteins present in cell lysates and sEVs, thus highlighting that they do represent their parental cell. However, this overlap is not complete, and specific pathways are enriched in sEVs, such as those involved in packaging sEVs. Interestingly, we also found the enrichment of proteins relevant in cancer, such as proteins involved in the PI3K-AKT pathway. These results highlight how sEVs can be used as a readout for processes in the parental cells, but, because certain pathways are enriched, the targets that are chosen as cancer biomarkers must be chosen with care. We hope that the data presented here can be an important reference for further research identifying potential cancer biomarkers from sEVs. 

## Data Availability

The data presented in this study have been deposited to the ProteomeXchange Consortium via the PRIDE partner repository with the dataset identifier PXD037651.

## Supplementary Materials

The following supporting information can be downloaded at: https://www.mdpi.com/article/10.3390/proteomes11010003/s1, Figure S1: Small-EV characterisation Western blot original blots. Figure S2: Replicates from shotgun proteomics cluster together with Pearson correlation and principal component analysis (PCA). Figure S3: Replicates from phospho-proteomics cluster together with Pearson correlation. Table S1: Proteomic abundances for CRC whole-cell preparation replicates. Table S2: Proteomic abundances for CRC small-EV preparation replicates. Table S3: Gene Ontology (GO) annotations for 531 proteins shared between small Evs from HCT116, HT29 and SW620 cell lines. Table S4: KEGG pathway enrichment analysis of HCT116, HT29 and SW620 using DAVID GO terms analysis for four different sets of proteins: (i) all proteins from whole cells, (ii) all sEV proteins, (iii) proteins observed in both whole cells and sEVs and (iv) proteins observed in sEVs only. Table S5: KEGG pathway analysis STRING enrichment for whole-cell and sEV proteins and sEV-specific proteins in HCT116, HT29 and SW620 cell lines. Table S6: Phosphopeptide abundances for CRC cell line whole cells. Table S7: Gene Ontology (GO) enrichment analysis of phosphoproteins represented in one cell line compared to the other two. Table S8: KEGG analysis of the differentially expressed phosphoproteins in one cell line compared to the other two.
