# Supplementary material for: Characterisation of Colorectal Cancer Cell Lines through Proteomic Profiling of Their Extracellular Vesicles
Source: Proteomes. 2023 Jan 11;11(1):3. doi: 10.3390/proteomes11010003 (PMC9844407; doi:10.3390/proteomes11010003)
Supplement: Supplementary file 1 [file proteomes-11-00003-s001.zip › Figure S1.pdf]

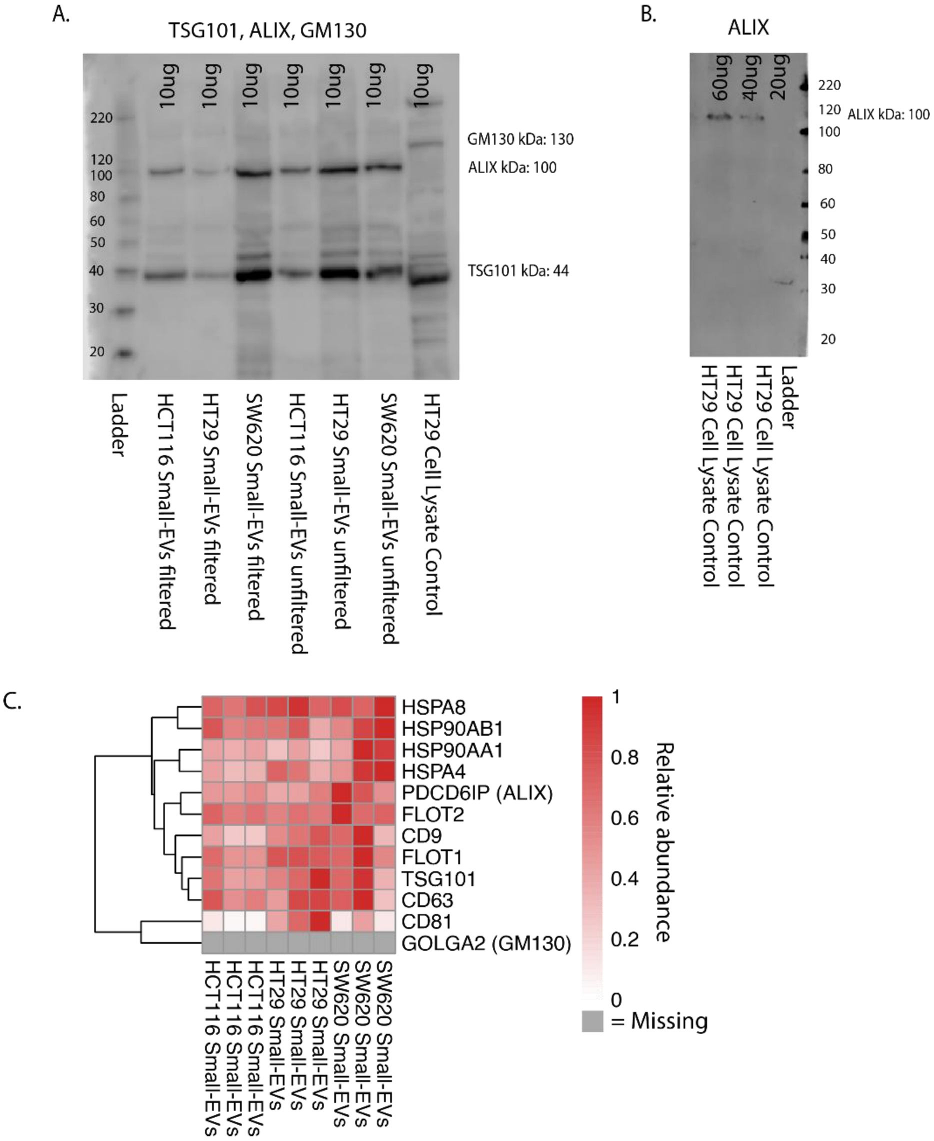

<u>Figure S1: Small-EV characterisation western blot original blots:</u> Original western blots of small-EV characterisation for HCT116, HT29 and SW620 cell lines using biomarkers TSG101, ALIX and GM130. (A) Shows the western blot for TSG101, ALIX and GM130 in small-EVs in both filtered and unfiltered samples. Filtered and unfiltered samples were being compared in isolation method establishment before settling on unfiltered small-EVs (image cropped accordingly in Figure 1B). Positive bands can be observed for TSG101 and ALIX in small-EVs (10ug loaded) in all cell lines and as expected, no bands are seen for small-EVs for GM130. A positive band for the HT29 cell lysate control is seen in TSG101 and GM130 when 10ug of protein is loaded. This is however not the case for ALIX with 10ug of protein. (B) Further ALIX blotting of the HT29 cell lysate control with larger amounts of protein show positive band for the cell lysate control from 40ug and 60ug protein. Unable to obtain such large amounts of protein for small-EVs although positive band noted at 10ug small-EV loading (Figure S1A). The image was cropped accordingly for use in Figure 1B. (C) Relative abundance of typical EV markers as well as the negative marker GOLGA2 as determined with mass spectrometry. Relative abundances are scaled to the sample with maximum abundance for each protein.
