# Supplementary material for: Characterisation of Colorectal Cancer Cell Lines through Proteomic Profiling of Their Extracellular Vesicles
Source: Proteomes. 2023 Jan 11;11(1):3. doi: 10.3390/proteomes11010003 (PMC9844407; doi:10.3390/proteomes11010003)
Supplement: Supplementary file 1 [file proteomes-11-00003-s001.zip › Figure S3.pdf]

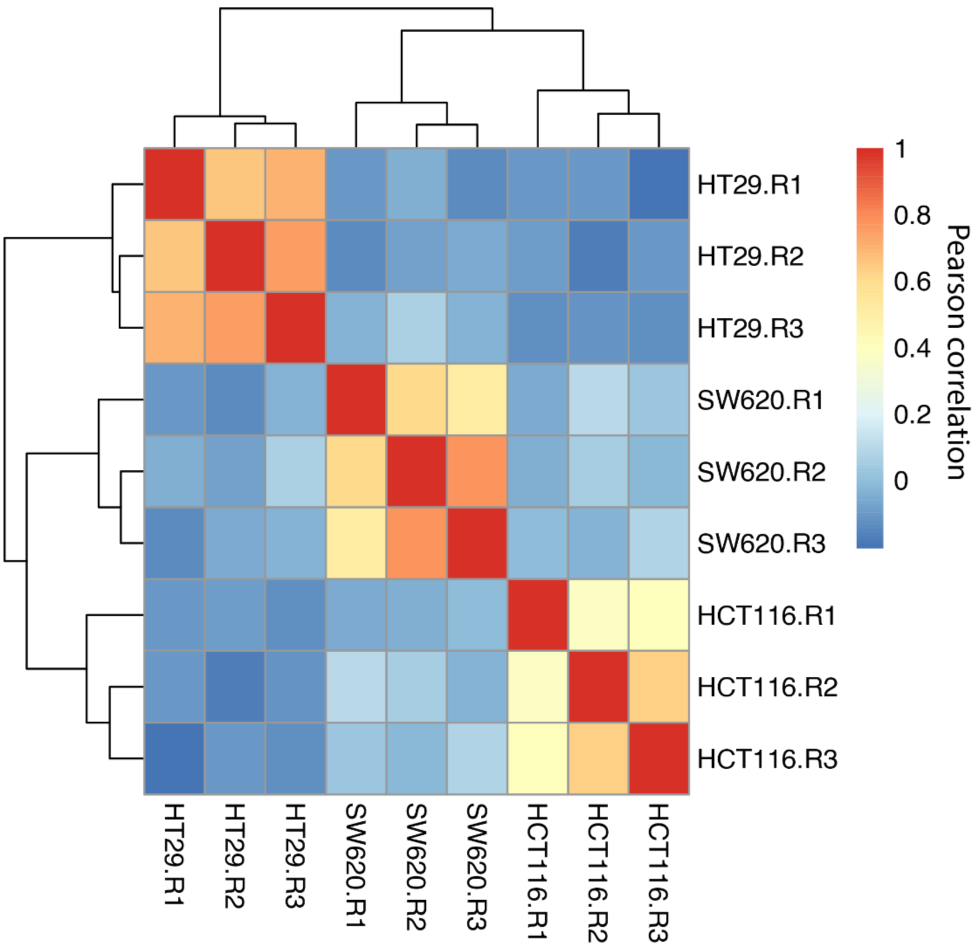

<u>Figure S3: Replicates from phospho-proteomics cluster together with Pearson correlation:</u> Heatmap showing Pearson correlation for all sample comparisons using scaled abundance values.
