# Supplementary material for: Characterisation of Colorectal Cancer Cell Lines through Proteomic Profiling of Their Extracellular Vesicles
Source: Proteomes. 2023 Jan 11;11(1):3. doi: 10.3390/proteomes11010003 (PMC9844407; doi:10.3390/proteomes11010003)
Supplement: Supplementary file 1 [file Figure S2.pdf › Figure S2.pdf]

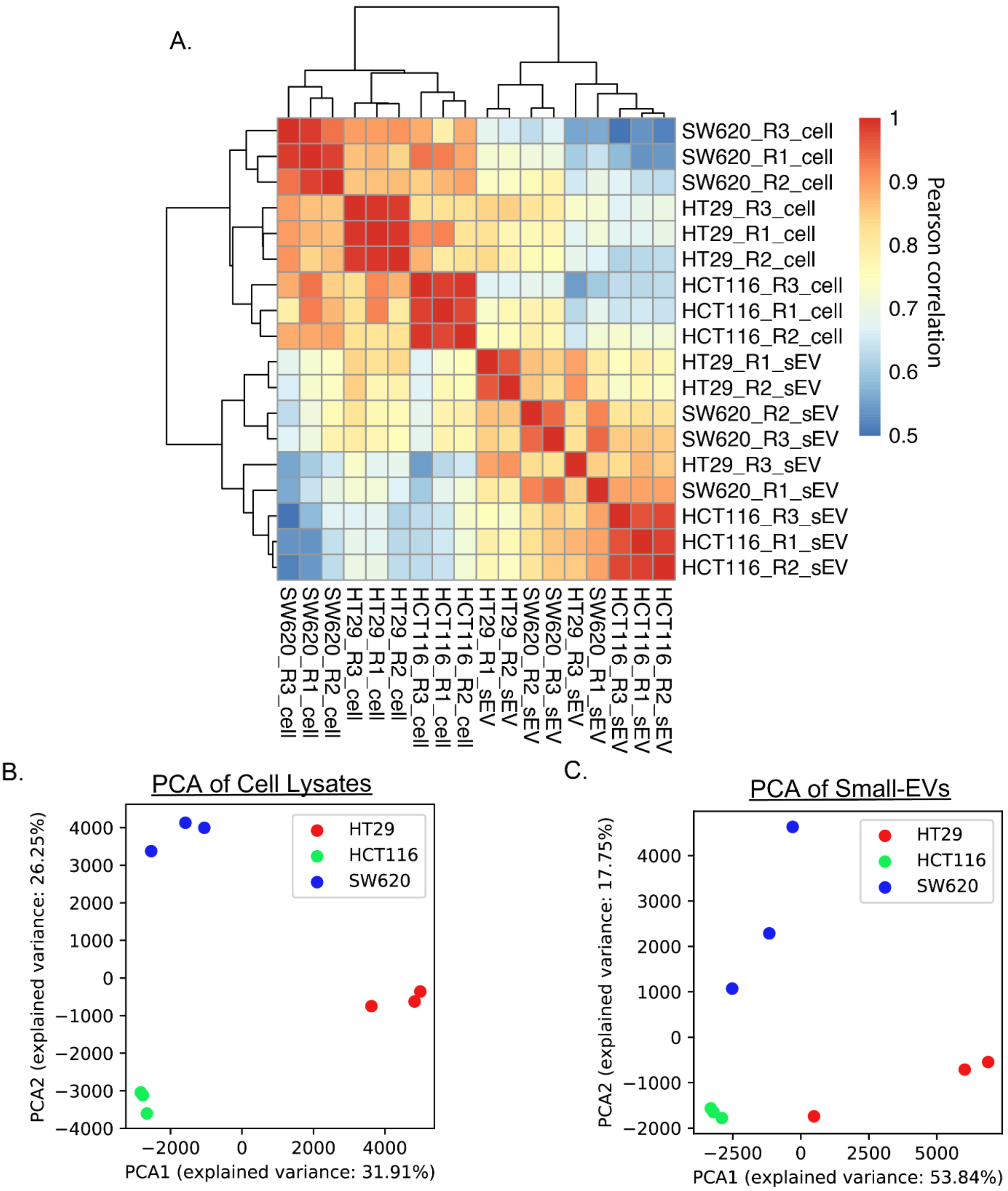

<u>Figure S2: Replicates from shotgun proteomics cluster together with Pearson correlation and Principal Component Analysis (PCA):</u> (A) Heatmap showing pearson correlation for all sample comparisons using the logarithm of normalized abundance values. (B) and (C) PCA for whole cell lysates (B) and small-EVs (C) displaying proteomic profile for the three replicas in each sample type.
